# P-491. Advancing HIV Care: Implementing Comprehensive Screening and Linkage Services in Diverse Urban Settings

**DOI:** 10.1093/ofid/ofae631.690

**Published:** 2025-01-29

**Authors:** Racquel Owino, Delana Gonzales, Steven Landin, Tanya Khalfan Mendez, Roseanne Lewis, Michelle Espinoza, Juan Bravo, Kimberlee Krull, Kasandra Johnston, Maria Hernandez, Tatiana Emanuel, Ralph Riviello, Anna Taranova

**Affiliations:** University Health, San Antonio, Texas; University Health, San Antonio, Texas; University Health, San Antonio, Texas; University Health, San Antonio, Texas; University Health, San Antonio, Texas; University Health, San Antonio, Texas; University Health, San Antonio, Texas; Bexar County Adult Detention Center, San Antonio, Texas; Bexar County Adult Detention Center, San Antonio, Texas; University Health, San Antonio, Texas; UT Health San Antonio, San Antonio, Texas; UT Health San Antonio, San Antonio, Texas; University Health, San Antonio, Texas

## Abstract

**Background:**

University Health is South Texas’ largest provider of specialty HIV/AIDS care to a 28-county region with an incidence rate of 21 per 100,000. San Antonio is designated as an HIV priority jurisdiction by the U.S. Department of Health and Human Services. University Health is suited to provide HIV services through its Emergency Department (ED), often the first point of care for indigent individuals, and screening at more than 19 primary care clinics and the Bexar County Adult Detention Center (BCADC).

**Methods:**

University Health implemented opt out HIV screening at three sites; 11 outpatient clinics, the ED, and the BCADC for adults 18 years and over following Centers for Disease Control and Prevention guidelines. EMR system prompts providers to order the preferred HIV confirmatory test using a Care Gap flag alert in outpatient clinics and a Best Practice Advisory in the ED. An HIV screening prompt on BCADC’s EMR system alerts providers to order and conduct an HIV rapid OraQuick swab. Upon identification of all confirmed diagnosis by a provider, the navigation team facilitates linkage to care.

**Results:**

From January 2023 to April 2024, 26,265 individuals were screened in the ED, identifying 205 as positive (0.8% seropositive). Among these, 34% were newly diagnosed, 64% were previously diagnosed, and 2% are pending diagnoses status. Among these, 57% of positive cases were linked to care. From January 2024 to April 2024, 2,415 individuals were screened in 11 ambulatory clinics, resulting in the identification of 3 positive cases (0.1 % seropositive), with 67% being new diagnoses and 33% being previously diagnosed. Of those with a positive result, 67% were linked to care. In the BCADC, spanning from February 2024 to April 2024, 2,473 individuals were screened, resulting in 20 positive cases (0.8% seropositive), with 70% being new diagnoses, 25% previous diagnoses, and 5% pending diagnoses status. Of those with positive diagnoses, 20% were linked to care.

**Conclusion:**

Multi-site expansion of HIV screening and linkage to care services promotes early detection, increased accessibility, and targeted interventions within a HIV priority jurisdiction. Universal screening aligns with national and global goals to end the HIV/AIDS epidemic.

**Disclosures:**

**All Authors**: No reported disclosures

